# Supplementary material for: Combined BSA-Seq and RNA-Seq Reveal Genes Associated with the Visual Stay-Green of Maize (Zea mays L.)
Source: Int J Mol Sci. 2023 Dec 18;24(24):17617. doi: 10.3390/ijms242417617 (PMC10744276; doi:10.3390/ijms242417617)
Supplement: Supplementary file 1 [file ijms-24-17617-s001.zip › ijms-2673733-supplementary.pdf]

Table S1. Quality statistics of mapping with the reference genome for BSA-Seq.

Sample	Read Sum	Base Sum	Q30(%)	GC percent(%)
T01	489.3M	73.3G	95.97	45.21
Xin3	465.8M	69.8G	96.19	46.66
H-pool	907.0M	136.0G	95.94	47.21
L-pool	802.0M	120.3G	96.04	47.40

Note: Q30(%): represented the percentage of bases with Phred value greater than 30 in the total base.

Table S2. The statistics of mapping results for RNA-Seq.

Sample Name	Total_Reads	Total_Bases	Q20%	Q30%	GC%
XC1	67966668	7739876821	98.79	96.04	55.60
XC2	66537680	7724550400	98.18	94.31	53.22
XC3	64830336	7348850800	98.98	96.79	51.63
XC4	62325672	7730652000	98.26	94.63	50.21
XC5	66715922	7695000200	98.54	95.34	51.33
XC6	68325699	7689633254	98.78	96.05	50.42
TC1	46474978	6832973378	98.93	96.50	51.23
TC2	43922438	6479543635	98.71	95.86	53.43
TC3	47122680	6694222958	99.08	97.02	50.72
TC4	32986302	4494076212	99.27	97.55	51.62
TC5	45399694	6589654569	99.02	96.79	51.01
TC6	42669454	6282347472	98.5	95.24	52.57
